# Speakers exhibit a multimodal Lombard effect in noise

**DOI:** 10.1038/s41598-021-95791-0

**Published:** 2021-08-18

**Authors:** James Trujillo, Asli Özyürek, Judith Holler, Linda Drijvers

**Affiliations:** 1 Donders Institute for Brain, Cognition and Behaviour, Radboud University Nijmegen, Nijmegen, The Netherlands; 2 Max Planck Institute for Psycholinguistics, Wundtlaan 1, 6525XD Nijmegen, The Netherlands; 3 Centre for Language Studies, Radboud University Nijmegen, Nijmegen, The Netherlands

**Keywords:** Human behaviour, Social behaviour

## Abstract

In everyday conversation, we are often challenged with communicating in non-ideal settings, such as in noise. Increased speech intensity and larger mouth movements are used to overcome noise in constrained settings (the Lombard effect). How we adapt to noise in face-to-face interaction, the natural environment of human language use, where manual gestures are ubiquitous, is currently unknown. We asked Dutch adults to wear headphones with varying levels of multi-talker babble while attempting to communicate action verbs to one another. Using quantitative motion capture and acoustic analyses, we found that (1) noise is associated with increased speech intensity and enhanced gesture kinematics and mouth movements**,** and (2) acoustic modulation only occurs when gestures are not present, while kinematic modulation occurs regardless of co-occurring speech. Thus, in face-to-face encounters the Lombard effect is not constrained to speech but is a multimodal phenomenon where the visual channel carries most of the communicative burden.

## Introduction

When communicating in face-to-face interactions, we often find ourselves in noisy situations, such as a cocktail party or a crowded restaurant. In these cases, our interactional partner may have trouble understanding what we are saying because of other speakers who are talking in the background. Previous research shows that in such noisy environments speakers automatically modulate auditory and visual (e.g., visual speech) features of their speech^1, 2^, an effect known as the Lombard effect. This modulatory increase in vocal effort includes an increase in speech intensity (i.e., loudness), and a shift in the fundamental frequency (F0; perceived as pitch). Similar to these auditory responses to noise, speakers also adapt the visual aspects of speech, such as lip movements^1, 2^. While this effect is partially reflexive^3^, both auditory^4, 5^ and visual^6, 7^ modulations are most prominent when an interactive partner is present. This suggests that the auditory and visual response to noise is at least partially a communicative adaptation designed for the listener.

Speech and accompanying lip movements are often considered the primary communicative signal, but they are heavily integrated with other signals from the face and body. Visual signals, including hand gestures^8^, are integral to human communication^9–12^. Especially co-speech representational hand gestures^11, 13, 14^ which can be described as the hand-movements that visually depict objects, actions, events, or spatial relations through the movements and configurations of the hands^8, 11^, may play a particularly important role in noisy situations when verbal communication fails^15^. Furthermore, similar to communicative adaptation in the Lombard effect, communicatively intended gestures produced without speech are larger and more temporally segmented when compared to the same gestures performed with no incentive to communicate^16^. These studies thus emphasize that, whether speech or gesture, communicative signals are adapted to the current communicative context^17, 18^. Transmitting messages successfully is the essence of human communication, and understanding how this is accomplished, especially in the natural, multimodal environment of language use^19^, is a crucial step in unravelling the workings of the human communication system. Consequently, a core question is whether interlocutors not only respond with speech-related adjustments but also gesturally to the presence of noise. Such evidence would necessitate a reconceptualization of the Lombard effect as a multimodal phenomenon that goes beyond speech and articulatory lip movements.

It is currently unknown whether and how speech and gesture are co-adapted to communication in noise. In terms of auditory and visual speech, previous research shows that speakers reduce their acoustic modulation (i.e., speech intensity) but increase visual modulation (i.e., mouth opening, or other facial kinematics) when their face is visible to the addressee^6^, indicating a shift in effort from the vocal to the visual modality. Conversely, there is evidence that speech and gesture often encode (semantic) information redundantly to increase communicative effectiveness^20, 21^. Redundant information encoding may extend to a redundant, or parallel, adaptation of gesture kinematics and speech acoustics. This follows from recent work showing an inextricable link between speech acoustics and gesture kinematics^22^. A more complete understanding of communicative multimodal adaptation requires us to determine whether speech and gesture are *both* modulated, and if so, if they are modulated independently of one another or with a shift of effort from speech to gestures, with gestures being the prime carriers of information.

Building on previous research, we ask whether and how communication in noise leads to a multimodal, communicative adaptation in speech, visual speech, and gesture. We first test whether the Lombard effect extends to gesture kinematics, and second, how the Lombard response differs as a function of whether speech or gesture are produced on their own or as one multimodal utterance. We used a relatively unconstrained dyadic interaction task with multi-talker babble played through the participants’ headphones to induce a Lombard effect. By combining this task with a non-traditional lab environment (i.e., a festival), we were able to elicit communicative behavior sampled from individuals with varied backgrounds, thus increasing generalizability of the data. We also use a novel paradigm for measuring the embodiment of the Lombard effect: namely, audio recordings accompanied by markerless motion tracking to assess the influence of noise on speech acoustics (i.e., auditory speech), face kinematics (i.e., visual speech), and gesture kinematics, as well as their interaction.

In sum, this study aims to advance our understanding of some of the basic principles that govern human communicative behavior in social, multimodal interaction. The findings elucidate how communicative demands can shape the acoustics and kinematics of multimodal utterances and extend previous models of speech-gesture production^23^. Specifically, Kita and Özyürek’s model^23^ postulates that utterances are planned by distributing information across speech and gesture according to linguistic formulation possibilities and communicative intentions. Our study builds on this model by defining the role of environment in influencing not only the content of an utterance, but also the form (i.e., kinematics and acoustics).

By examining specific kinematic features, we can show how the form of a gesture is modulated. For example, spatial features, such as the relative height or size of a gesture, could be enhanced to make the signal more salient, similar to what is seen in mouth opening size in visual speech. Alternatively, the temporal structure of a gesture could be modified to make it more salient (i.e., increased peak velocity) or more clearly segmented (i.e., more holds between movements, or larger number of distinct movements). Enhancing movement boundaries, as would be evident from more holds per gesture, directly relates to Garnier and colleagues’ finding that content-word boundaries are enhanced in Lombard speech^24^. Finally, the overall amount of visual information can be increased, either through more repetitions of a key movement (e.g., a hammering motion can be repeated any number of times) or through a more complex representation of an action (e.g., using one hand to depict placing a nail, then the other to hammer, as opposed to only depicting the hammering motion), which would be seen as an increase in gesture submovements. This would be in line with findings that repetition^25^ and complexity^26^ are used when depicting novel, relevant information, and would suggest that a similar kinematic strategy is used to highlight gestural information when communicating in noise.

To this end, we employed a dyadic interaction task with multi-talker babble played through headphones worn by both participants to induce a Lombard effect. We used three noise levels in order to determine whether the multimodal signal was differentially adapted to the amount of noise. We maintained the communicative context throughout the experiment by asking one participant to communicate a number of action verbs to the other participant in a spontaneous manner. We use audio recording and markerless motion tracking to assess the influence of noise on speech acoustics (i.e., auditory speech), face kinematics (visual speech), and gesture kinematics, as well as their interaction with one another. We further focus our analyses on *communicative attempts* (i.e., spoken utterances, gestures, or multimodal [speech + gesture] utterances separated by at least 200 ms from any other communicative behavior) in order to zoom in on the interaction between the vocal and visual modalities.

When using unimodal communicative acts (speech or gesture alone), we expect enhancement in the respective signal (speech = acoustic and/or visual, gesture = visual), replicating studies of speech production, and extending previous notions of gestural enhancement. In terms of multimodal communicative attempts, one hypothesis (*the parallel-enhancement-hypothesis*) is that when speech is enhanced, gesture follows suit, with both modalities contributing to a potentially more robust signal^20^. This hypothesis would suggest signal-level modulation is implemented as a global modulation, separate from or in parallel with content-formulation. An alternative hypothesis (*the flexible-enhancement-hypothesis*) is that the visual and vocal signals work together but that each can be flexibly enhanced without necessarily affecting a parallel change in the other to meet current communicative demands^27^. This alternative hypothesis would suggest that signal-level modulation is built into the planning of how information will be distributed across speech, visual speech, and gesture. Finally, in order to determine how environmental constraints such as noise influence the relative weight of speech or gesture we ask whether there is a preference for multimodal utterances over unimodal ones in increased noise.

## Results

### Effect of noise on speech acoustics

Speech acoustic analyses utilized maximum speech intensity and F0 values, calculated per *communicative attempt* (i.e., spoken utterances, gestures, or multimodal [speech + gesture] utterances separated by at least 200 ms from any other communicative behavior; see “Methods” and Supplementary Material). The speech analyses only utilized communicative attempts that actually included speech, and the number of speech-only and multimodal utterances used in these analyses is provided with the results.

To assess whether noise level affected speech acoustics, we fitted two linear mixed models, one predicting speech intensity, the other F0. Models included noise as an independent variable, as well as random terms for participant and item (i.e., the word being described) and when possible, a random slope to account for interindividual differences in the effect of noise on speech acoustics. These models were compared against a null model that contained the random terms, but not noise level.

Speech acoustic analyses were based on data from 243 Speech-only communicative attempts and 327 Multimodal attempts (Table 1). No random slopes were included in these models due to singular fits.

**Table 1:** Overview of modality usage (total frequency of attempts, and percentage of all attempts per noise level) across noise levels.

	Modality			Total
	Speech only	Gesture only	Multimodal	
8-Talker babble	94 (21.5%)	244 (55.8%)	99 (22.6%)	437
4-Talker babble	71 (17.3%)	252 (61.8%)	88 (21.4%)	411
Clear	78 (17.2%)	236 (51.9%)	140 (30.8%)	454
Total	243	732	327	

**Figure 1:**
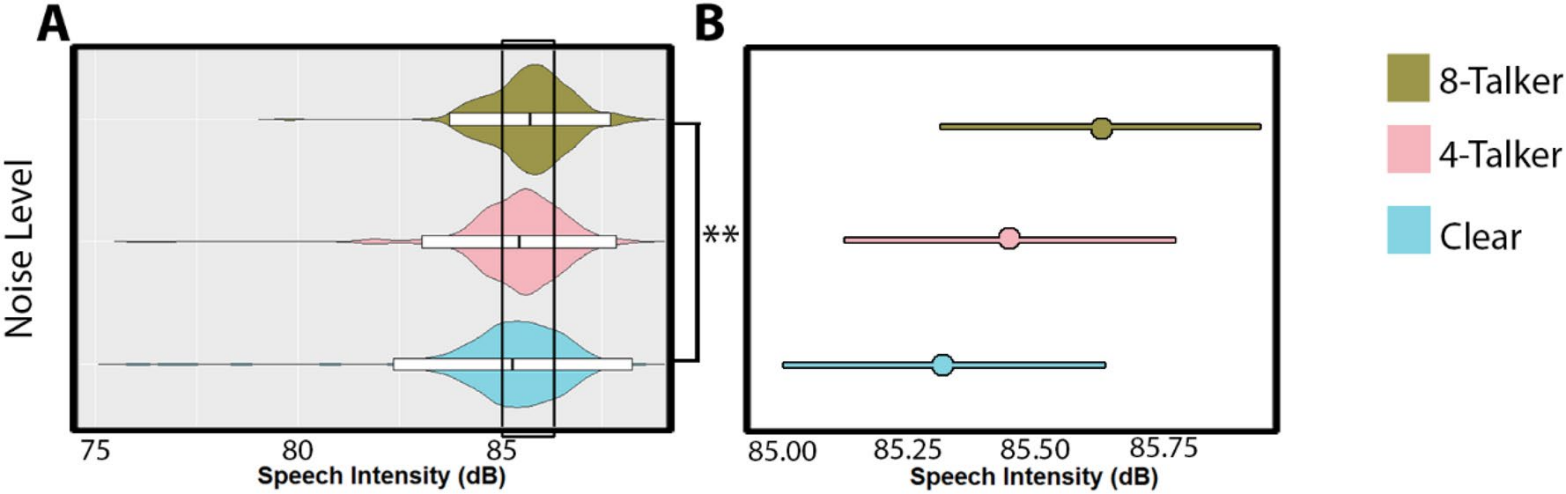
Overview of significantly affected speech acoustics across noise levels. Panel (**A**) depicts the raw distribution of Speech Intensity values, where the y-axis shows the three noise levels in ascending order, while the x-axis shows the raw acoustic values. Violins represent the kernel probability of the data at each point. Within each violin a boxplot shows the mean (middle bar) and two standard deviations from the mean (box hinges). Panel (**B**) depicts the model estimates for Speech Intensity. In other words, the predicted values of Speech Intensity based on noise level, while holding all other random terms constant. The central circle provides the mean, while the bars on either side provide the confidence intervals based on standard error. Significant contrast comparisons between noise levels are indicated by a bracket. **p < 0.001.

Noise level in these attempts was strongly associated with speech amplitude, χ^2^(2) = 11.49, *p* = 0.003. Specifically, 8-talker babble was associated with an increase of 0.29 ± 0.09 dB compared to no noise (t-ratio = 3.37, *p* = 0.002), while no association was found between 4 t-talker babble and no noise (t-ratio = 1.32, *p* = 0.387), and no significant difference was found between 8-talker and 4-talker babble (t-ratio = 1.82, *p* = 0.164). Noise level was not significantly associated with F0, χ^2^(2) = 1.685, *p* = 0.431. See Fig. 1 and Table 1 for an overview of these distributions.

### Effect of noise on face kinematics

Face kinematic analyses utilized maximum mouth opening, mean lip movement (i.e., mean frame-by-frame displacement of the lower lip), and peak lip velocity (i.e., maximum velocity achieved by the lower lip), calculated per *communicative attempt*. As with the speech analyses, the face kinematic analyses only utilized communicative attempts that included speech, and the number of speech-only and multimodal utterances is provided with the results.

To assess whether noise level affected face kinematics, we fitted linear mixed models for each kinematic feature. Models included noise as an independent variable, as well as random terms for participant and item (i.e., the word being described) and when possible, a random slope to account for interindividual, as well as inter-item differences, differences in the effect of noise on face kinematics. These models were compared against a null model that contained the random terms, but not noise level.

Face kinematic (i.e., visual speech) analyses were based on data from 243 Speech-only attempts and 327 Multimodal attempts (Table 1). Max mouth opening and mean lip movement included random slopes for participant. Noise level was significantly associated with maximum mouth opening, χ^2^(7) = 33.88, *p* < 0.001 as well as mean lip movement, χ^2^(7) = 16.62, *p* = 0.020, but not with peak lip velocity, χ^2^(2) = 5.11, *p* = 0.078. For both maximum mouth opening and mean lip movement, post-hoc comparisons revealed no significant differences between the individual noise levels in terms of mean kinematic values. In order to qualitatively assess whether the lack of significant contrasts is due to wide interindividual variation in the direction of effect (i.e., positive versus negative association), we include a visualization of the effect of noise on face kinematic values, plotted per participant, in Supplementary Figure 1. The slopes indicate that while there was a relationship between noise and face kinematics, for some participants this was a negative relationship, while for others it was positive.

In order to determine whether facial kinematic effects could be explained by the increase in speech acoustics alone, we carried out an additional, exploratory analysis that tested whether a model with noise and speech intensity as fixed effects is a better fit than a model with only speech intensity. If the model is a better fit with noise than without, this would suggest that the facial kinematic effects are not only being driven by speech acoustics, but are indeed an (at least partially) independent response to noise. Results showed that maximum mouth opening is significantly better explained by noise and speech intensity than by speech intensity alone, χ^2^(2) = 34.48, *p* < 0.001. Mean lip movement was not better explained by the addition of noise, χ^2^(2) = 0.392, *p* = 0.822.

### Effect of noise on gesture kinematics

Gesture kinematic analyses utilized holdtime (i.e., amount of time the hands do not move during a gesture), submovements (i.e., number of constituent movements), maximum distance (i.e., size of the gesture), peak velocity (i.e., maximum velocity achieved by either hand involved in the gesture), and vertical amplitude (i.e., maximum height achieved by either hand involved in the gesture), calculated per *communicative attempt*. Gesture kinematic analyses only utilized communicative attempts that included gesture, and the number of gesture-only and multimodal utterances is provided with the results.

To assess whether noise level affected gesture kinematics, we fitted linear mixed models for each kinematic feature. Models included noise as an independent variable, as well as random terms for participant and item (i.e., the word being described) and when possible, a random slope to account for interindividual, as well as inter-item differences, differences in the effect of noise on gesture kinematics. These models were compared against a null model that contained the random terms, but not noise level.

**Figure 2:**
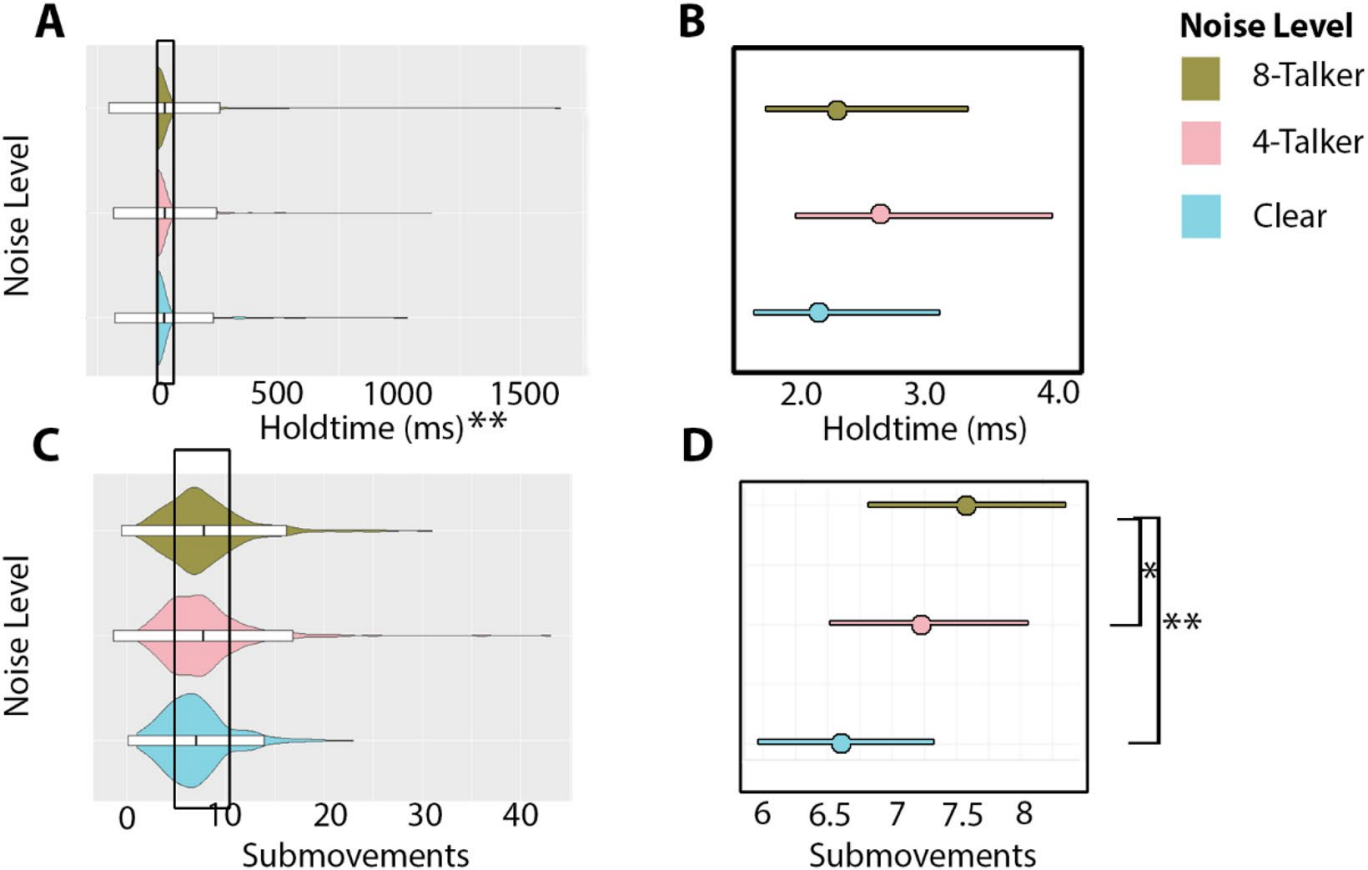
Overview of significantly affected body kinematics across noise levels. Panel (**A**) depicts the raw distribution of holdtime values, while panel (**B**) depicts the model estimates for holdtime. Panel (**C**) provides the raw distribution of submovement values, while panel (**D**) depicts the model estimates for submovements. In panels (**A**) and (**B**), the y-axis shows the three noise levels in ascending order, while the x-axis shows the raw kinematic values. Violins represent the kernel probability of the data at each point. Within each violin a boxplot shows the mean (middle bar) and two standard deviations from the mean (box hinges). In panels (**C**) and (**D**), the predicted kinematic values are based on noise level, while holding all other random terms constant. The central circle provides the mean, while the bars on either side provide the confidence intervals based on standard error. Significant contrast comparisons between noise levels are indicated by a bracket. The significant main effect of Holdtime is indicated by asterisks on this plot. *p < 0.05. **p < 0.001.

Data were based on 732 Gesture-only and 327 Multimodal attempts (see Table 1). Only holdtime included both a random intercept and random slope for participant**.** Noise level was positively associated with the number of submovements, χ^2^(2) = 22.519, *p* < 0.001. Specifically, 8-talker babble showed 1.142 times the number of submovements as the clear condition (t-ratio = 4.68, *p* < 0.001), and 4-talker babble showed 1.093 times the number of submovements as the clear condition (t-ratio = 3.01, *p* = 0.007). We additionally found a significant relationship between noise and holdtime, χ^2^(7) = 179.74, *p* < 0.001. Post-hoc comparisons revealed no overall significant positive or negative effect of any noise level on holdtime. This indicates that, similar to maximum mouth opening and mean lip movement, this feature is highly variable between participants. We found no difference between 8-talker babble and 4-talker babble (t-ratio = 1.51, *p* = 0.398). There was a marginally significant effect of noise on maximum distance (i.e., gesture size), χ^2^(2) = 6.16, *p* = 0.046. Noise level showed no association with peak velocity, χ^2^(2) = 0.01, *p* = 0.998, nor with vertical amplitude (4-talker > clear: z = -0.708, *p* = 0.479; 8-talker > clear: z = -1.228, *p* = 0.219). See Fig. 2 for an overview of significant kinematic effects.

### Interaction between speech and gesture

**Figure 3:**
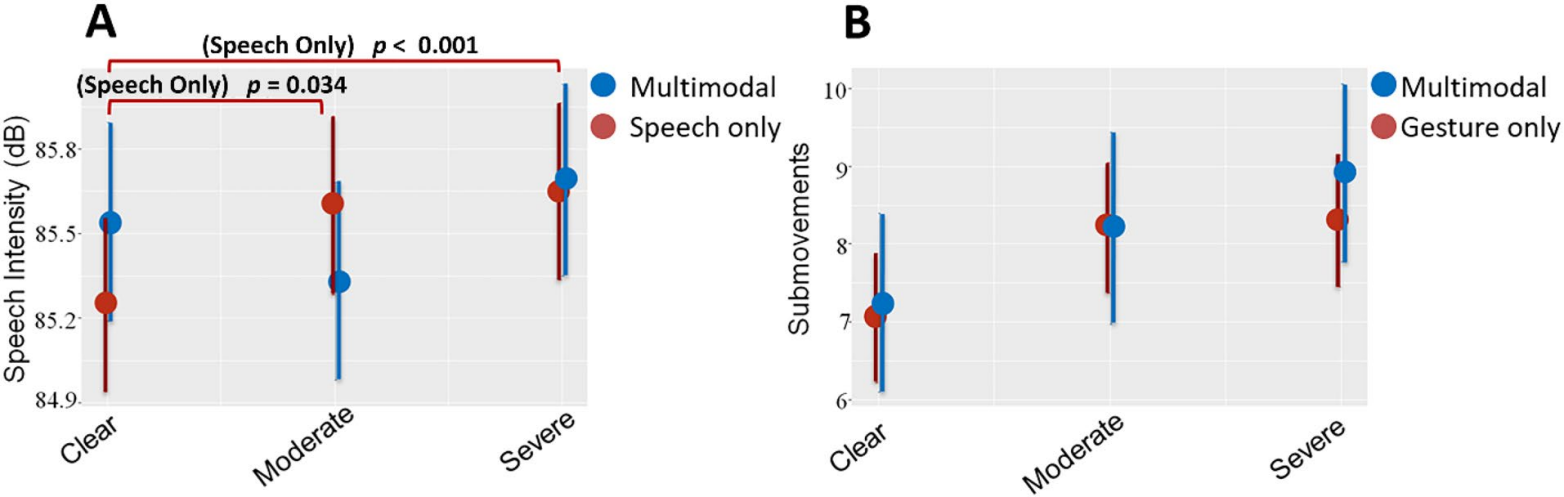
Speech intensity and gesture submovements across noise levels, as produced in unimodal or multimodal utterances. (**A**) Depicts speech intensity (y-axis), with blue lines representing multimodal (speech + gesture) utterances and red lines depicting unimodal (speech only) utterances. (**B**) Depicts submovements (y-axis), with blue lines representing multimodal (speech + gesture) utterances and red lines depicting unimodal (gesture only) utterances. In both plots, the three noise conditions are plotted on the x-axis. Circles represent the mean of the distribution, while line lengths extend to ± one standard deviation. p-values are provided for significant comparisons. Unmarked comparisons are non-significant.

In order to test whether the signal modulations in speech and gesture occur in both unimodal and multimodal or only in unimodal utterances, we assess whether there was an interaction effect between noise level and modality (i.e., unimodal or multimodal). We found no interaction between noise and modality for submovements, χ^2^(3) = 0.666, *p* = 0.881, holdtime, χ^2^(3) = 2.669, *p* = 0.446, maximum mouth opening, χ^2^(3) = 6.464, *p* = 0.091, or mean lip movement, χ^2^(3) = 1.776, *p* = 0.620. For speech amplitude, we found a significant noise × modality interaction, χ^2^(3) = 11.78, *p* = 0.008. Post-hoc comparisons showed that speech-only utterances had a significant increase in speech amplitude in both 8-talker babble compared to clear (estimate = 0.427, t = 4.152, *p* < 0.001) as well as in 4-talker babble compared to clear (estimate = 0.275, t = 2.496, *p* = 0.034). However, we found no effect of noise on speech amplitude in multimodal utterances. In other words, speech seems to be flexibly modulated based on the presence or absence of gestures, while gesture is modulated any time they are used (see Fig. 3).

Finally, we used a cumulative link mixed effects model to test whether increasing noise level led to a greater frequency of multimodal utterances compared to unimodal (speech-only or gesture-only). We found no evidence for more multimodal utterances in 8-talker babble (z = 1.809, *p* = 0.071) or in 4-talker babble (z = − 0.005, *p* = 0.996) compared to clear. See Table 1 for the distribution of modality across noise levels.

## Discussion

Gesture is a crucial component of everyday human communication. Yet, many aspects of language use are investigated primarily as unimodal phenomena consisting of speech alone. The Lombard effect is one such phenomenon: a plethora of studies has convincingly demonstrated that, in the presence of noise, speakers enhance their vocal communication (such as through louder utterances and wider mouth openings^2, 4, 7^. However, how the gestural signals that accompany speech in face-to-face settings, contributing crucially to semantic communication, are involved in this process had never been investigated. Thus, the aim of this study was to determine how *multimodal* human communication is adapted to adverse situations, such as noise.

Our primary interest was in determining whether the Lombard effect extends to gesture kinematics. Second, we aimed to determine whether speech, visual speech, and gesture are all enhanced in noise (i.e., parallel enhancement hypothesis) or if there is a shift towards either auditory or kinematic enhancement (i.e., flexible-enhancement hypothesis). Our results confirm that the Lombard effect is indeed a multimodal phenomenon, with increasing noise modulating not only speech acoustics and facial kinematics (i.e., visual speech) but also—and in many cases only—gesture kinematics. In terms of communicative strategy, speakers either raise the intensity of their voice to overcome the noise when not using gestures, or they take advantage of the visual channel, such as gestures, to communicate *without* putting extra effort into the auditory signal. This demonstrates a primary role for visual kinematic enhancement, with flexible-enhancement of the acoustic signal.

Although our study focuses on multimodal communication, it was important to first establish the classic Lombard effect in speech in order to understand how gesture fits into the bigger picture of multimodal adaptation. The most universal finding is an increase in vocal intensity in response to noise^28^, which we replicated here. The lack of increase in F0 was initially surprising, but can be explained by the overall high vocal intensities observed across all noise levels. Although F0 typically increases together with intensity^29^, F0 levels saturate at high levels, such as during shouting or loud speech^30^. In loud speech, as we observe in the current study, there may be variation in speech intensity, but little or no variation in F0. This is evidenced in the mean vocal intensity values (Figs. 1, 3), which are similar to values reported for shouted speech^31^. Overall, however, we can conclude that our experiment successfully elicited the Lombard effect, replicating the effect in a more ecologically valid environment than ever before.

Using a novel, sophisticated motion tracking approach, we found evidence for an effect of noise on facial kinematics. At least in the case of mouth opening size, this effect could not be explained by the enhanced speech acoustics alone. However, the directionality of this effect seemed to differ between participants. This suggests that some individuals responded to noise by making their visual speech more salient with larger mouth opening and overall more lip movement, while others may have reduced or otherwise neglected to change their facial kinematics. This finding is directly in line with that of Garnier and colleagues^7^ who likewise found that different participants respond differently in terms of visual speech adaptation in response to noise. Given that there may be a shift from vocal to visual adaptation in audio-visual Lombard speech^32^, our findings suggest that exaggeration of facial kinematics is a very personal strategy. Overall, our findings support the hypothesis that facial kinematics are adapted to noise, at least by some individuals.

Critically, in addition to a modulation of speech signals, we found that gesture submovements were increased in noise compared to no noise. By focusing our analyses on single communicative attempts we can be confident that this effect is capturing the amount of visual information being conveyed in a single gesture. In other words, the effect is unlikely to be due to non-communicative artefacts such as false starts (i.e., preparing a gesture and then abandoning it before producing the full gesture) or repairs (such as refashioning a gestural depiction), since these would have not been coded (in the case of the former) or have been coded as separate gestures (in the latter case). Instead, submovements characterize the amount of segmented visual information being presented through the hands. This can be through the number of gesture strokes being produced, or the extent to which a complex gesture expression is segmented and repeated in clearly defined individual movements. For example, a gesture can be elaborated by adding a new representation, such as first making a ‘dipping’ motion to represent dipping a chip in sauce, then depicting the eating of the chip. A gesture can also show repetition by repeating the same action or concept in a slightly different space, or simply with a pause in between. Although fully assessing this distinction in submovement function is beyond the scope of this paper, manual coding of a subset of our data (17%) revealed that 43% of instances of multiple gestures within one communicative attempt are explained by an elaboration, while repetitions make up the remaining 57%. While this is not statistically analyzed, it provides an idea of the function of submovements. These descriptions can be found in Supplementary Table 4. Thus, we can demonstrate that the increase in submovements is likely due in large part to repeating a salient component of the gestural depiction, although in many cases it may indeed arise from elaborations of a representation. This is in line with gesture studies suggesting that more elaborate, demonstrative gestures are used when in dialogue compared to monologue^33^, and when the gestural referent is more communicatively relevant^34^. Similarly, repetitiveness is a characteristic of child-directed-action^25, 35^. In other words, repetition and elaboration are established strategies to make visual depiction more salient.

The additional finding of holdtime being affected by noise, at least in some individuals, directly mirrors the findings of Garnier and colleagues^24^, who similarly found that some participants increase speech boundaries in noise. Here, we show that temporal segmentation of movement boundaries is also performed by some individuals in response to noise. Overall, the finding of kinematic modulation in response to noise is directly in line with the idea that movement kinematics (i.e., the way a gesture is produced) are adapted to the communicative context in which they are produced^16, 26, 27, 36^.

Our findings provide a nuanced answer to the question of how noise affects the interplay between speech and gesture in face-to-face communication. We find that the contributions of the specific signals (i.e., speech, gesture) can be flexibly adapted to the needs of the current situation, with the visual signals potentially carrying most of the communicative burden. More generally, participants favored gesture-only utterances, suggesting a primary role for adapting the gestural modality to noise, with flexible adaptation of the acoustic (i.e., speech) signal. When the utterance is unimodal, utilizing speech only, then acoustic modulation is required in order to increase the signal-to-noise ratio of the speech signal, in line with past research^37^. However, when it is multimodal, it appears not to be necessary to put more effort into the speech signal as gestures and visual speech help clarify speech in noise^15, 38^. This fits well with recent findings demonstrating, from the side of comprehension, that the impact of multimodal cues depends on the informativeness of all other co-occurring signals^15, 39^.

Models of multimodal language production have suggested that information is distributed across the visual and vocal modalities according to representational and linguistic constraints^23^. Our results demonstrate that it is not only the content of speech and gesture that is distributed and specified, but also the signal-level characteristics (i.e., kinematics and acoustics) that are specified during this phase. It should be noted that the current findings only show how the producer responds to noise. While we know that gestures and facial information support comprehension of speech in noise^15^, we cannot say anything about the communicative effectiveness of kinematic adaptation in this context. Future studies should further develop these models to include the dynamical interaction between producer and addressee in order to gain a more complete understanding of how these signal-level characteristics are further modulated by interaction.

Studies on the Lombard effect typically compare a nearly entirely noise-free situation with a situation in which there is a disruptive amount of background noise. In many real-life situations, this distinction is not so clear. It is therefore interesting to see that although speech intensity values were high throughout the experiment, likely due to the ambient noise of the festival environment, there was still evidence for a gestural Lombard effect**,** individual-specific visual speech modulation, and a flexible modulation of speech acoustics in response to the experimental noise levels. This demonstrates the propensity of the gestural modality to respond to environmental noise with an enhanced gestural signal, and the robustness of this multimodal Lombard effect.

One limitation of our study is that the specific set-up of the experience may have influenced participants’ behavior. For example, the distance between participants may have encouraged participants to use more gestures and less speech, whereas this may be less necessary when people stand closer together. Similarly, while the environment of the experiment (i.e., a music festival) provided a unique sample of participants, it may also have contributed to additional influencing factors. One example is the use of alcohol by participants in the hours before the experiment. Although participants were instructed not to have consumed alcohol in the preceding hour, any drinking during the rest of the day could have influenced their behavior. However, an additional statistical check showed that self-reported alcohol intake did not significantly contribute to the observed effects (see Supplementary Material). Despite these limitations, the within-subjects design, allowing us to see that these participants adjusted their vocal and gestural behavior in response to changes in noise level, suggest that these results are still robust. Future studies should replicate these findings in a more controlled laboratory setting in order to disentangle any potential confounding factors.

The present study shows that multimodal communication, at the level of acoustics and kinematics, is flexibly adapted to the setting in which it is produced. Results demonstrate the visual Lombard effect extends beyond visible speech and into gestures. The visual component may be the more prominent communicative adaptation, given that face and gesture kinematics are modulated in both multimodal and unimodal utterances. In contrast, acoustics are less enhanced when speech is paired with gestures. This suggests that noise affects the dynamics of the speech-gesture system, likely at the level of communicative planning. These findings demonstrate that the Lombard effect should be reconceptualized as a multimodal phenomenon that affects speech and gesture production differently depending on which signals are used.

## Methods

### Participants

Data were collected at *Lowlands Science*, a science-outreach focused event taking place at the Lowlands music festival in Biddinghuizen, The Netherlands on August 17–19, 2018. We tested 91 pairs of participants, resulting in an initial sample size of 182 participants. We excluded participants for whom there was incomplete data or technical problems during acquisition, participants who were intoxicated by drugs or alcohol, and those who were overly familiarized with the stimuli. This was possible because the experiment was public, therefore participants could watch others completing the experiment. We limited our analyses to the first participant (the Producer) in each pair (see subsection Paradigm). This led to a total sample size of 58 native Dutch participants (32 females, 20 males, 6 missing; mean age 27.75 ± 7.9 years). Fifty-four were right handed. For the four left-handed participants, we focused on left-handed kinematics (see Supplementary Material for more info). This study was approved by a local ethics committee (CMO region Arnhem-Nijmegen, the Netherlands) and all participants gave informed consent regarding use of their behavioral data. The study was conducted according to the guidelines of the Declaration of Helsinki. Images depicting participants used in this manuscript are from participants who additionally gave informed consent for their images to be published in open access scientific publications.

### Set-up

Participants took part in the experiment in pairs, with one starting as the "Producer" and the other starting as the "Addressee". The two participants were separated by a one-way screen that reduced visibility of the Addressee to the Producer, but allowed the Addressee to see the Producer. This reduced the amount of feedback that the Producer could receive, and thus the extent to which their behavior could be affected by not only the communicative context, but by the ongoing feedback of their Addressee. In order to be able to draw firm conclusions about the effect of noise on gesturing, we aimed to reduce the influence of addressee feedback (which has been shown to affect the form of gestures by itself, in the absence of noise) on gestures. The experiment area was visible to the public, but observers were not allowed to come close enough to interfere or disrupt the experiment. While this could have allowed some priming of behavior, most participants did not wait long before beginning the experiment and were mostly occupied with filling in participant background forms. Given that the experiment took place over many hours and across multiple days, we also believe that any potential priming effects would not be substantial. Using four lists of words for the task (see below) additionally assured that participants were not overly primed with the words that they would be given, even if they had seen part of the previous participants’ session.

**Figure 4:**
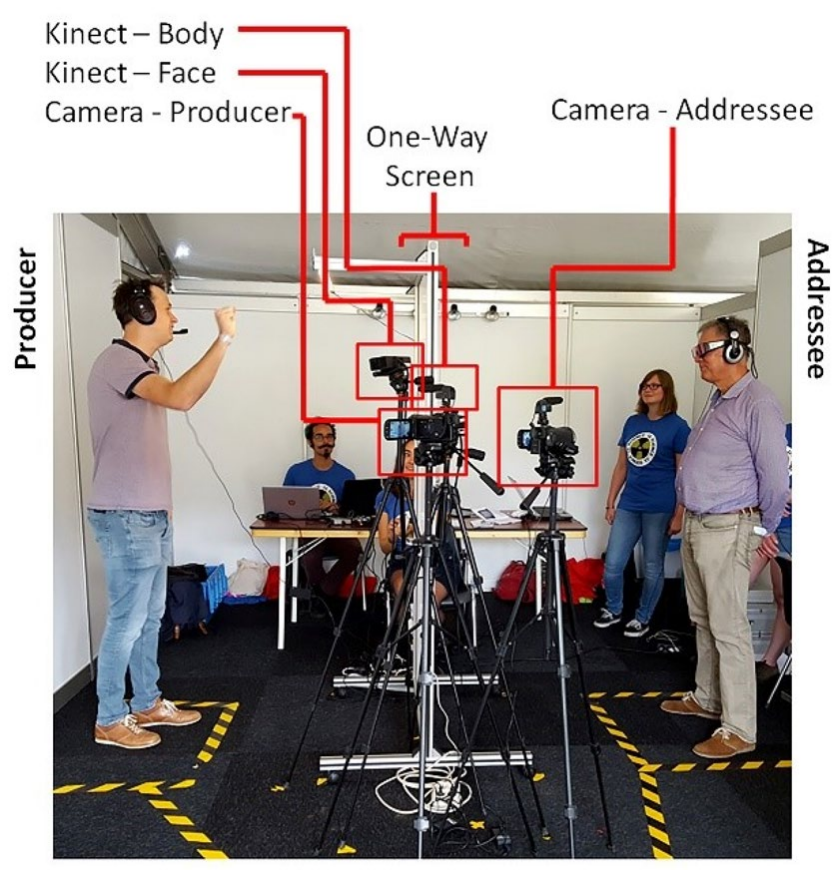
Overview of the physical set-up of the experiment. The producer can be seen on the left side, while the Addressee can be seen on the right side. A one-way screen separates them, allowing the addressee to see the producer, but obscuring the Producer's view of the addressee. Two Kinects (one for face tracking and one for body tracking) are directed at the Producer. One video camera is facing the Producer, while a second video camera faces the Addressee. The yellow and black markings on the floor indicate the area in which the participants must remain throughout the experiment.

Both participants wore noise-cancelling headphones through which they heard multi-talker babble, a type of pre-recorded audio in which speech from multiple speakers are overlaid on top of each other, thus simulating noise similar to that of a noisy cocktail party or restaurant (see Supplementary Material for more info). The addressee always heard 4-talker babble, while sound in the Producer's headphone varied randomly from round (i.e., word) to round between clear (no noise), 4-talker babble, and 8-talker babble. This allowed us to test whether signal modulation was dependent on the specific noise level, or a general response to the presence of noise. Producers were informed that the addressee would always hear the same amount of noise, while their own noise level would vary from round to round. Both participants were informed that the one-way screen would result in vision being obscured for the producer, but not for the addressee. Producers were recorded using microphones attached to their headphones, two Microsoft Kinects, and one video camera, all positioned approximately one meter away from the Producer, positioned at head-height directly next to the one-way screen. The Kinect is a markerless motion-tracking device that uses a depth camera to capture three-dimensional video, from which key-points on any person within frame are detected using a software implementation. This allows one to track the body (i.e., One Kinect was used to track whole body motion, while the second was used to track the face). The addressee was recorded using a video camera, positioned one meter away, directly next to the one-way screen. The Addressee additionally wore eye-tracking glasses (see Fig. 4). However, given the focus of this study was on the Producer, we will not discuss data or results from the Addressee.

### Task

The task of the Producer was to communicate individual verbs to the Addressee. Each participant completed a list of 20 different action verbs. Verbs were selected based on pretesting that ensured they were all high-frequency words that did not differ in terms of recognizability or potential for gestural depiction (see Drijvers and Özyürek, 2017 for more details). Four different lists were used, with participant pairs always having separate lists, and the following pair having the remaining two lists (see Supplementary Table 1 for the lists of words). The word was presented by one of the experimenters using large white flashcards with the word printed in black. Producers were instructed that they should attempt to communicate the word in any way they wanted, but were not given any specific instructions or suggestions as to how they should do this. Producers could therefore speak, gesture, or use any combination of movement and speech. We chose not to explicitly instruct or manipulate modality usage in order to avoid potential confounding effects of instruction on the communicative signals that we were interested in^40^ and understand how modalities are spontaneously employed in the context of noise**.** Each round ended either when the Addressee correctly verbally identified the word (successful), or when too much time (approximately one minute) had passed (unsuccessful). Feedback was given as a "thumbs-up" or "thumbs-down" gesture by the experimenter to indicate the round was complete, either successfully or unsuccessfully. Noise level varied randomly from each round, or verb, to the next. Thus, no two words were repeated within a single participant, and any interaction between specific words and noise level was controlled for by random assignment.

### Annotation of communicative attempts

Communicative attempts were defined as being a single attempt to communicate the target word. This could be unimodal or multimodal, and could contain multiple gestures or speech utterances. All videos were manually annotated in ELAN^41^. Attempts were distinguished from one another based on temporal proximity and communicative strategy.

Individual gestures were identified and annotated at the level of ‘gesture units’, which include the preparation, stroke, and retraction^42^. In other words, a gesture was annotated as the complete set of preparation, stroke, and retraction, when these phases were available. This was done in order to capture all kinematic features relating to the complete gesture unit. False starts were not annotated as gestures, while repairs (e.g., refashioning a gesture after starting) were annotated as separate gestures, and separate communicative attempts, as long as the repair occurred after the stroke phase. For the purpose of finding communicative attempts, only representational gestures^8, 11^ were used. Representational gestures are any gestures that visually depict or refer to actions, people, objects, or ideas. This may be, for instance, through acting out an action (i.e., pantomiming), or tracing or molding an object shape.

For speech, we only used speech utterances containing the target word. Speech utterances could be a single word, or a longer utterance. Utterances were annotated based on semantic and pragmatic coherence, without taking co-occurring gestures into consideration (see Supplementary material for more information).

Unimodal utterances could be gestures with no temporally overlapping speech, or speech utterances with no temporally overlapping gesture. Communicative attempts could therefore be speech-only, gesture-only, or multimodal (speech and gesture with temporal overlap) (see Supplementary Figure 2). For the purpose of this study, we only utilize data from the first communicative attempt in each round. This was done to obtain as ‘clean’ a dataset as possible, because subsequent attempts will likely represent an evolution of communicative strategy in response to feedback. Table 1 provides an overview of the distribution of modality use, within the first attempt, across the noise levels. More information about the annotation procedure is provided in the Supplementary Materials section.

### Feature calculation

#### Speech acoustics

**Figure 5:**
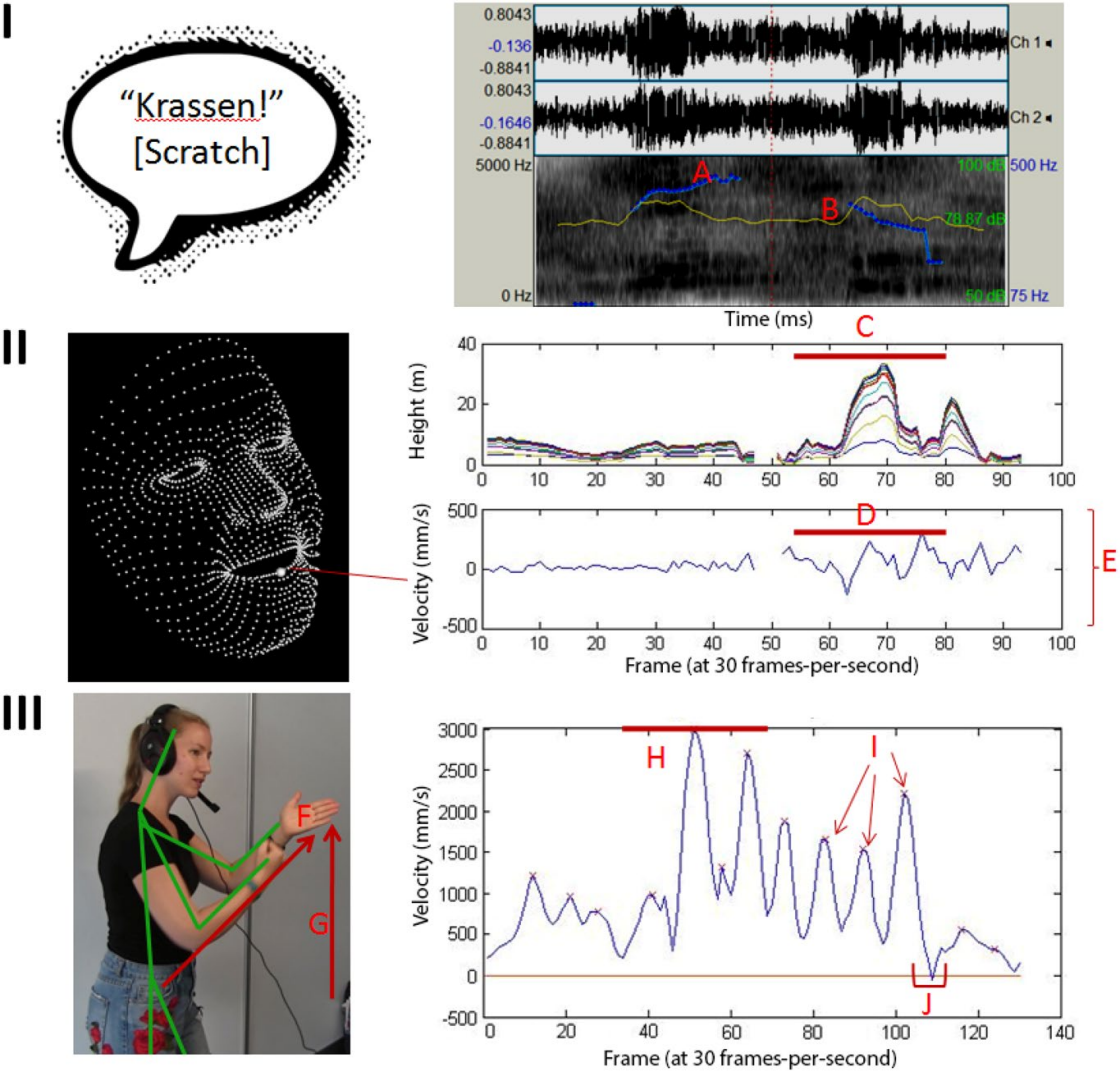
Graphical overview of all communicative features calculated. Panel (**I**) depicts the speech acoustics, with upper right plot (taken from PRAAT) showing the speech waveform together with the intensity and pitch envelopes. Panel (**II**) depicts the face kinematics. The tracked points of the face are displayed on the left, with emphasis on the middle lower lip point that was used for the peak velocity calculation. The right plots of panel (**II**) show the mouth opening and lip movement through time. Panel (**III**) depicts the gesture kinematics. The graphic on the left is a still frame from the same communicative utterance from which the kinematic plots are derived, with an overlay of the Kinect tracking lines. The right bottom plot shows the velocity profile of the right hand. (**A**) F0 is given as the blue line. (**B**) Speech intensity is represented by the yellow line. (**C**) Mouth opening is the highest value within one attempt by any two pair of points. (**D**) Peak lip velocity was the highest velocity achieved by the lower lip. (**E**) The overall amount of movement of the lower lip, taken as the average per second. (**F**) Maximum distance of the hand from its starting point. (**G**) Vertical amplitude of the hands. (**H**) Peak velocity of the hand. (**I**) Number of submovements, visible here as individual peaks. (**J**) Holdtime, seen here as the amount of time spent below the velocity threshold.

For speech, we calculated maximum intensity (i.e., loudness, in dB) and maximum F0. Both features were calculated at the word or longer utterance level (depending on the length of the attempt made), rather than syllable level. Maximum intensity and *F0* were calculated using PRAAT^43^ (see Fig. 5, panel I). Intensity and F0 were selected as they are the most prominent features of Lombard speech and are related to communicatively-motivated enhancements. We used the onset and offset time of individual utterances (i.e., words or phrases uttered without pause) within each attempt to calculate these features, rather than taking the entire communicative attempt. This was done to ensure that we only captured the acoustics of the communicative speech that was annotated.

#### Face kinematics

For the face tracking data, we only calculated features in attempts that included speech. We calculated the *Maximum Mouth Opening* by taking the distance between all tracked points (see Supplementary Material for information about motion tracking) corresponding to the inner area of the mouth and finding the maximum value per communicative attempt. We calculated *Mean Lip Movement* as the average distance traveled by the lips per second. Finally, we took the peak velocity achieved by the center point of the bottom lip in order to give the *Peak Lip Velocity*. These variables represent an enhancement of the visual speech signal through spatial (mouth opening) and temporal (velocity) increases, while total movement captures the amount of visual information being presented. Note that we took the peak velocity in order to correspond with the hand kinematic measures. These features were based on previously established visual features of the Lombard Effect as described by Heracleous et al. (2013) and Kim et al. (2005) (see Fig. 5, panel II).

#### Gesture kinematics

For the body tracking data, we calculated kinematic features using the toolbox developed in Trujillo, Vaitonyte et al. (2019). In sum, we calculated *peak velocity* of the dominant hand as the highest velocity achieved during the attempt, which can characterize gesture effort. *Vertical amplitude* characterized the maximum height achieved by either hand in relation to the body (0—below middle of torso; 1—middle of torso to upper quarter of torso; 2—within upper quarter of torso; 3—above shoulders; 4—above middle of face; 5—above head). *Holdtime* represents the total amount of time, during gesture production, during which the hands were still, thus segmenting the gesture and drawing attention to the position or configuration of the hands. *Submovements* represent the number of individual movements made by the dominant hand, which characterizes the amount of visual information being presented, such as the number of stroke (i.e., the semantically meaningful part of the gesture) repetitions, and/or the number of different movements being made within one gesture. Increasing submovements and/or holdtime would indicate that participants are packing more well-segmented visual information into their gestures. For example, increased submovements can relate to more repetitions of a key movement (e.g., a hammering motion can be repeated any number of times) or a more complex representation of an action (e.g., using one hand to depict placing a nail, then the other to hammer, as opposed to only depicting the hammering motion). We used these kinematic features as they have been related to a higher intention to communicate^16^, as well as to the semantic comprehension of gestures^44^. We additionally calculated *maximum distance* as the maximum distance away from the body achieved by the dominant hand. This feature was added in order to include an additional purely spatial kinematic feature (see Fig. 5, panel III). Increasing vertical amplitude or maximum distance would indicate that participants are trying to compensate for the disruption of the auditory signal by making the visual signal more salient through increasing the size of the gesture. This set of features were calculated for the time-window of individual gesture occurrences within the first communicative attempt.

### Analysis

All statistical analyses were carried out using the R statistical program (R Core Team, 2019).

#### Effect of noise on speech and gesture

Before proceeding with statistical analyses, we tested all dependent variables (kinematic and acoustic features) for multicollinearity by calculating the variance inflation factor as described by Zuur and colleagues (Zuur, Leno, and Elphick, 2010). Predictors with a variance inflation factor greater than three were excluded from all subsequent analyses (see Supplementary Table 2).

We used linear mixed-effects models to calculate the influence of noise on each of our dependent variables. Mixed-effects models were implemented using the lme4 package^45^. We created nine generalized linear mixed-effects models, each with one of the features of interest (submovements, hold-time, peak velocity, maximum distance, maximum mouth opening, lip movement, lip velocity, speech intensity, speech F0) as the dependent variable, with noise level as a fixed-effect, and a random intercept for each participant and item. To test the significance of these models, we used Chi-square difference tests to compare the models of interest with a null model. As different words may lead to differences in kinematic or acoustic features, we first tested whether a null model containing both participant and word as random intercepts was a better fit to the data than a model with only participant as random intercept. The better fitting model was used as the null model against which the kinematic and acoustic models were tested. We additionally included random slopes for each random term in the model, in keeping with the guidelines of Barr and colleagues^46^. In the case of a singular model fit, the slope for the random effect with the lower explained variance was dropped. If both slopes led to singular fit, we used a model without random slopes. After finding the maximal random effects structure, we again used Chi-square model comparison to determine if this more complex model was a better fit to the data than the null model.

When appropriate, generalized linear mixed models were used together with either Poisson or gamma distributions. We performed this step for variables that are better described as a count of events (i.e., Poisson distribution for submovements) or time accumulation (i.e., gamma distribution for hold-time). We determined that these generalized mixed models were a better fit by visually inspecting the model residuals with a standard Gaussian distribution compared to the alternative Poisson or gamma distribution as well as using Chi-square model comparison between the two. This approach has been advocated to robustly deal with skewed or variable data while maintaining interpretability of results (Lo & Andrews, 2015). This led to submovements being modeled with a Poisson distribution and holdtime being modeling with a gamma distribution. This approach allowed us to keep the full variability of our data, rather than removing outliers^47^. However, to ensure that outliers were not exerting undue influence on our results, we additionally ran our analyses with values beyond the mean + 3 standard deviations removed. These analyses revealed the same pattern of effects as the models that included all data points (see Supplementary Table 3 for an overview of all final models).

Due to the ordinal nature of vertical amplitude, we used a cumulative link linear mixed effects model, implemented with the R package “Ordinal”^48^, rather than the linear model for this aspect of the gesture kinematic analyses. In keeping with the linear mixed models, participant and word were included as random terms in the model. For cumulative link mixed models, significance is determined by directly assessing model parameters, rather than using Chi-square tests model comparisons.

#### Differential effects of noise on unimodal versus multimodal responses

Our second research question was whether noise leads to a general (parallel) increase in all signals or a flexible, signal-specific adaptation. In order to test whether there was an interaction between modality (i.e., speech without gesture or gesture without speech, compared to speech + gesture attempts) and noise level, we added an interaction term into any acoustic or kinematic model for which there was a main effect of noise, leading to a model that included terms for modality, noise, and modality × noise as fixed effects. Pairwise comparisons were calculated with estimated marginal means, with Tukey’s correction for multiple comparisons, as implemented with the Emmeans package^49^. Second, we tested whether participants were more inclined to use multimodal responses in the presence of more noise, which would determine whether there was a more coarse-grained response, beyond signal modulation. This was done with a cumulative link mixed model, with noise level as the predictor variable and modality (unimodal or multimodal) as the dependent variable, and participant and item as random intercepts.

## Supplementary Information

Supplementary Information.
