# Supplementary material for: Speakers exhibit a multimodal Lombard effect in noise
Source: Sci Rep. 2021 Aug 18;11:16721. doi: 10.1038/s41598-021-95791-0 (PMC8373897; doi:10.1038/s41598-021-95791-0)
Supplement: Supplementary file 1 — Supplementary Information. [file 41598_2021_95791_MOESM1_ESM.docx]

**Supplementary Material**

*Participant and technical set-up*

Participants were not selected in any way, but volunteered for participation in this experiment. Festival goers can freely enter the Lowlands Science terrain to participate in a number of socio-psychological experiments. All participants were tested between noon and 8pm across three consecutive days.

In terms of alcohol consumption, 24 had no alcohol on testing day, 24 had between one and three beverages, six had between four and six beverages, and four had six or more beverages. We obtained ethics approval from the Ethics Assessment Committee of the Faculty of Arts and the Faculty of Philosophy, Theology and Religious Studies (EACH) of the Radboud University Nijmegen prior to the festival. Participants were required to give informed consent to the use of their data for scientific research prior to participating, with the option to give informed consent for use of images and videos in publications and/or popular media. Participants did not receive any financial compensation. Prior to participating, we collected information regarding participants' age, gender, hand-preference, number of alcoholic beverages consumed on the day of the experiment, and whether any drugs were used on that day. Four participants indicating have used drugs in the past 24 hours.

Multi-talker babble stimuli were created by overlaying recordings of storytelling in Dutch, with half of the speakers in each condition being male and half female Noise volume for both participants was manually adjusted by the experimenter to achieve the highest volume that participants could tolerate without being painful.

*Data and Processing*

Video

Video was recorded at 25 frames per second (fps) with audio sampling at 44,100Hz. The Kinect tracked the face and body at 30fps. Kinect data for the body consisted of 25 tracked joints. Analyses specifically used the middle of the spine, shoulders, elbows, wrists, and center of each hand. Tracking for the face consisted of 1,324 points, constituting a mesh that covered the entire face. Our analyses utilized the points corresponding to the lips.

Kinect

Kinect data was used to calculate a set of kinematic features describing movements of the face and hands (see subsection Feature Calculation). All motion tracking data was smoothed using a Savitsky-Golay filter with a span of 15 and degree of 5 to correct for artefacts in the tracking. All data smoothing and kinematic features were calculated using MATLAB 2015a (The MathWorks, Inc., Natick, Massachusetts, United States) (see Trujillo, Vaitonyte, Simanova, & Özyürek, 2019). Due to the relatively unconstrained nature of the task we chose to focus on the first communicative attempt (see subsection Annotation of Communicative Attempts) within each round. This ensures that we capture the initial response to the specific noise condition as well as the first multimodal utterance, before it is affected by repetitions or changes in communicative strategy. Therefore, all features are calculated for single attempts, rather than single gestures or entire rounds. Similarly, the first time a target word is spoken during an attempt, this utterance is used to calculate acoustic features. In each feature, for both acoustics and kinematics, we removed all outlying data points that were more than 1.5 times the interquartile range away from the median.

Annotation

For gestures, we used the description of a ‘gesture unit’ provided by Kita, van Gijn and van der Hulst (1998), which includes the preparation phase, stroke phase, and retraction phase. One gesture annotation could thus maximally include each of these phases, and minimally include a stroke, which is the most semantically meaningful part of the gesture. For example, if one stroke is followed by a preparation phase for a different stroke, the first gesture is annotated up to the end of the first stroke phase, and the second is annotated beginning at the preparation of the second gesture. Gestures that were aborted before completing the stroke phase, such as false starts that lead to a self-repair or change in strategy, were not considered part of a communicative attempt. Repairs that occurred after a completed stroke, thus constituting a revision or change of strategy, were annotated as separate gesture events, and separate communicative attempts. For this study, we focused on representational gestures, which are those gestures that visually depict actions, object, or abstract ideas. Our analyses thus excluded other gesture types, such as interactives that are meant to indicate to the addressee that they are thinking (e.g. hands out, palms up), or emblems such as the thumbs-up gesture. While these are interesting in their own right, we chose to focus our analyses on the meaning-bearing parts of speech and gesture.

For the purpose of defining communicative attempts we did not consider speech that was motivational rather than informative (e.g. "not quite...", "come on"), or speech that was not directed to the addressee (e.g. "hmm, okay"). Speech utterances were annotated such that if the target word was uttered in isolation, it was considered a single utterance, but if the target word was uttered in the context of a complete phrase/phrases, then the phrasal context was considered part of the attempt (e.g. for target word “climb”, one could utter “(you) climb up a mountain”).

In the case of speech or gesture immediately preceding the other, these cases were considered to be one attempt when there were five or fewer video frames (approximately 200ms or less) between the onset of one and offset of the other. In the case of multiple gestures, these were considered to be part of one attempt when there were two or fewer frames between them, or if there was no full retraction. In the case of three or more frames between gestures, or a complete retraction, even if this occurs in fewer than three video frames, these gestures are considered to be part of two separate communicative attempts. Two speech utterances were considered part of separate attempts if there more than five frames (approximately 200ms) between them.

The 200ms threshold was used for two speech or two gesture occurrences because this is believed to be the minimally perceptible pause duration in conversation (Walker & Trimboli, 1982) and has previously been used for segmenting speech utterances (Heldner, Edlund, Hjalmarsson, & Laskowski, 2011). The three-frame difference for determining whether speech and gesture were part of one utterance was based on perceptual studies of speech-gesture integration, which we used to approximate when the two signals were likely to be perceived as one coherent utterance (Habets, Kita, Shao, Özyurek, & Hagoort, 2010). This provides a relatively conservative measure determination of speech-gesture utterances. These rules can be summarized as follows:

Unimodal: Gesture

No overlapping speech

No speech within 5 frames

No complete retraction gesture and speech onset

Multi-gesture: onset must be less than 3 frames from previous gesture offset

Unimodal: Speech

No overlapping gesture

No gesture (with incomplete retraction) within 5 frames

Multi-speech: onset must be less than 5 frames from previous speech utterance

Multimodal

Speech and gesture temporally overlap, or

Speech and gesture no more than 5 frames apart

In case of gesture preceding speech, there must be no complete retraction of the gesture

After the initial gesture and speech segmentation, another annotator coded ten of the video files, approximately 20% of the data, to check for coding reliability. We calculated a modified cohen’s kappa, following the procedure of Holle and Rein ( 2015). For speech, we obtained a raw agreement of 95.3% and a kappa of 0.952, while for gesture we obtained a raw agreement of 85.9% and a kappa of 0.746. These values represent substantial near perfect agreement for speech and substantial agreement for gesture (Landis & Koch, 1977), and thus a good reliability of the coding.

*Analysis*

In order to account for potential correlations between kinematic features, and between acoustic features, as well as the increased type-I error rate associated with multiple comparisons, we used Simple Interactive Statistical Analysis (http://www.quantitativeskills.com/sisa/calculations/bonfer.htm) to calculate an adjusted Bonferroni correction using the mean correlation between the tested features. This mean correlation, and thus the adjusted alpha threshold, was calculated separately for body kinematics, face kinematics, and acoustic features. This is due to the fact that each of these signals represents a separate family of tests. In other words, we are not testing whether noise has an effect on specific features, but whether noise affects speech acoustics, face kinematics, or body kinematics. Body kinematics showed a mean correlation of 0.135, leading to a Bonferroni corrected alpha threshold of 0.0124. Face kinematics showed a mean correlation of 0.286, leading to a Bonferroni corrected alpha threshold of 0.031. Speech acoustics showed a mean correlation of -0.066, leading to a Bonferroni corrected alpha threshold of 0.026.

For all speech acoustic models, the model containing both participant and verb as random intercepts was used as the null model. For face kinematics, maximum mouth opening and peak lip velocity used null models with participant as random intercept, while mean lip movement used a null model with both participant and verb as random intercepts. For all gesture kinematic models, the model containing both participant and verb as random intercepts was used as the null model.

In order to provide some indication of what an increase in the number of movements may indicate, we have checked the manual annotations of a subset of our data (10 participants, 17% of the data), and assessed when there were multiple separable gesture strokes within the same communicative attempt. This is a more coarse-grained assessment of gesture movements, as the submovements feature captures every movement making up even a gesture stroke. For example, a single stirring gesture can be broken down into each circular motion of the hand (i.e., each individual submovement). However, by looking at the manually-annotated gesture strokes, we hope to provide a more easily interpretable, coarse view of the way participants may be adding information to their communicative attempts. We therefore found each instance in this data subset where a participant had more than one gesture stroke per attempt, described what each stroke is representing, and denoted whether subsequent strokes could be considered an elaboration (i.e., adding a new representation, such as first making a ‘dipping’ motion to represent dipping a chip in sauce, then depicting the eating of the chip), or a repetition (i.e. representing the same action or concept in a slightly different space, or simply with a pause in between). This information is provided in the appendix to this letter, and can also be provided as an additional appendix to the manuscript. In short, we find that elaborations make up approximately 43% of these data, while repetitions make up the other 57%.

*Effect of Alcohol on Main Outcome Measures*

In order to ensure that alcohol did not influence our results, we performed an additional step in our model fitting. This was done to assess whether including alcohol (as a self-reported number of drinks a participant has had in the last 24 hours) in our final models provided a better fit than the final model without alcohol.

We find no evidence for alcohol contributing to submovements (χ^2^(1)*=*2.603, p = 0.107), holdtime (χ^2^(1)*=*0.6773, p=0.411), mouth opening ( χ^2^(1)=0.631, p = 0.427), lip movement (χ^2^(1)*=*0.009, p=0.927), or speech intensity (χ^2^(1)=0.02, p=0.887).


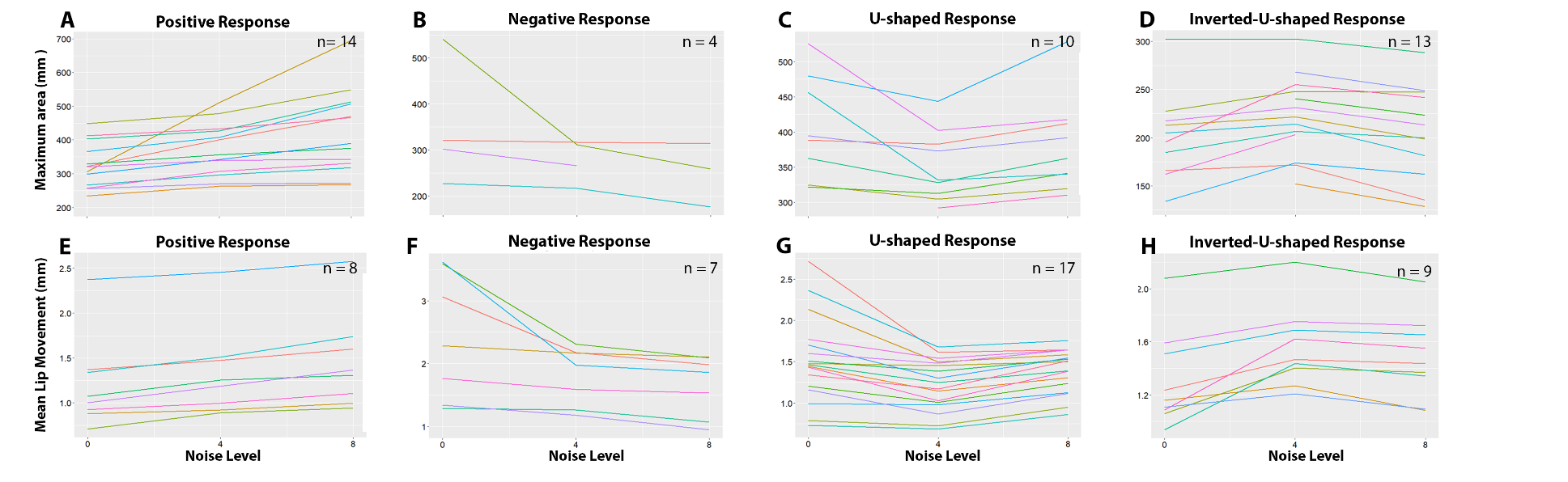
Supplementary Figure 1. Relationship between noise level and face kinematics across individuals. Panels **A-D** depict max mouth opening in mm^2^, while panels **E-H** depict mean lip movement in mm. Panels A and E show participants who exhibited increased kinematic values in response to noise, panels B and F show participants who exhibited decreased kinematics in response to noise, panels C and G show participants with a U-shaped response, and panels D and H show participants with an inverted-U-shaped response. In all plots, the three noise levels are given along the x-axis while the kinematic values are given along the y-axis. In the upper right corner of each plot, the number of participants contributing to that plot is given. Each participant is represented by a different colored line.


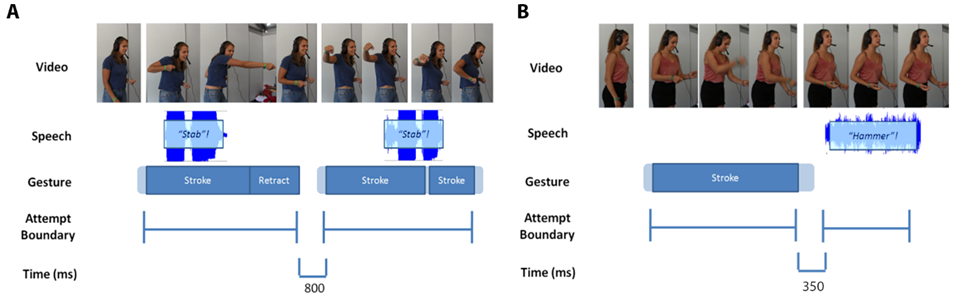


Supplementary Figure 2. Examples of attempt coding, and unimodal versus multimodal attempts. Both examples depict rounds with two communicative attempts. In both panels, the top panel, Video, provides Still frames from the corresponding video. Speech, depicts the coded speech, overlaid on the speech waveform. Gesture shows the individual gesture strokes and, when present, retractions. Attempt Boundary shows how the two attempts were defined. Time shows the (rounded, for simplification) number of milliseconds between the two attempts. In A, the first attempt is considered multimodal, due to the temporally overlapping speech and gesture. The attempt is finished after the gesture, as there is full retraction. The second attempt is also multimodal, but this time with two gesture strokes as well. The two strokes belong to the same attempt both because they occur with the same speech utterance, but also due to the close temporal proximity, as visualized by the overlap in the opaque blue bar behind the Gesture coding. In B, both attempts are unimodal. The first attempt is gesture only. After the gesture stroke, there is no retraction, but it is followed by a speech utterance. As there are more than 200ms between the two, they are counted as two attempts.

| **rollen** | to roll | **beklimmen** | to climb | **roeren** | to stir | **ophangen** | to hang up |
| --- | --- | --- | --- | --- | --- | --- | --- |
| **dobbelen** | to roll dice | **bidden** | to pray | **hameren** | to hammer | **uitrekken** | to stretch out |
| **strijken** | to iron | **drijven** | to float | **afgieten** | to drain | **boren** | to drill |
| **afdrogen** | to dry off | **filmen** | to film | **filmen** | to film | **klappen** | to clap |
| **slaan** | to hit | **stempelen** | to stamp | **beklimmen** | to climb | **schudden** | to shake |
| **drummen** | to drum | **raspen** | to grate | **bidden** | to pray | **zagen** | to saw |
| **verplaatsen** | to move | **skiën** | to ski | **drijven** | to float | **zouten** | to salt |
| **opzoeken** | to look for | **boksen** | to box (sport) | **vangen** | to catch | **aankruisen** | to cross |
| **tekenen** | to draw | **bladeren** | to page through | **wijzen** | to point | **hakken** | to chop |
| **wassen** | to wash | **slingeren** | to swing | **dippen** | to dip | **plukken** | to pluck |
| **toetsen** | to honk | **verbinden** | to connect | **groeien** | to grow | **stapelen** | to stack |
| **smeren** | to smear | **wenken** | to beckon | **poolen** | to play pool | **pellen** | to peel |
| **snijden** | to slice | **krassen** | to scratch | **vlechten** | to braid | **vissen** | to fish |
| **zwemmen** | to swim | **weggooien** | to throw away | **breien** | to knit | **toeteren** | to honk |
| **plakken** | to stick | **kietelen** | to tickle | **verschuiven** | to push | **ritsen** | to zip |
| **darten** | to throw darts | **schrijven** | to write | **jojoën** | to yo-yo | **fietsen** | to bicycle |
| **ontkurken** | to uncork | **steken** | to stab | **indrukken** | to push in | **omdraaien** | to turn around |
| **verdelen** | to divide | **schrobben** | to scrub | **opkloppen** | to beat | **knippen** | to cut |
| **schroeven** | to screw | **kloppen** | to knock | **hijsen** | to hoist | **sluipen** | to sneak |
| **golven** | to play golf | **timmeren** | to nail | **rijden** | to ride | **mixen** | to mix |

Supplementary Table 1. List of all verbs used in the experiment. Words in **bold** are the original Dutch words as they were used, while the English translations are given in the next column to the right.

| **Variable** | **Variance Inflation Factor** |
| --- | --- |
| *Gesture* | |
| Max distance | 1.415811 |
| Submovements | 1.184686 |
| Holdtime | 1.022674 |
| Vertical Amplitude | 1.107519 |
| Peak Velocity | 1.212722 |
| *Face* | |
| Max Mouth Opening | 1.096874 |
| Mean Lip Movement | 1.010711 |
| Peak Lip Velocity | 1.089116 |
| *Speech* | |
| Speech Intensity | 1.017859 |
| Speech F0 | 1.017859 |

Supplementart Table 2. Variance Inflation Factors (VIF) for all independent variables.

| **Final Model** | **Noise**  **β** | **Noise**  **t-ratio** | **Noise**  **p** | **Model comparison**  **Χ^2^** | **Model comparison *p*** |
| --- | --- | --- | --- | --- | --- |
| **MaxDistance** ~ noise + (1\|PAIR)+(1\|Verb) |  |  |  | 0.698 | 0.705 |
| **Submovements** ~ noise + (1\|PAIR)+(1\|Verb),  family=Poisson |  |  |  | 22.519 | < 0.001 |
|  | *8-talker – clear* | | |  |  |
|  | 0.133 | 4.679 | <0.001 |  |  |
|  | *4-talker – clear* | | |  |  |
|  | 0.089 | 3.013 | 0.007 |  |  |
|  | *8-talker – 4-talker* | | |  |  |
|  | 0.044 | 1.509 | 0.287 |  |  |
| **PeakVelocity** ~ noise + (1\|PAIR)+(1\|Verb) |  |  |  | 5.266 | 0.072 |
| **Holdtime** ~ noise + (1+noise\|PAIR),  Family=Gamma |  |  |  | 179.74 | <0.001 |
|  | *8-talker – clear* | | |  |  |
|  | -.031 | -0.442 | 0.898 |  |  |
|  | *4-talker – clear* | | |  |  |
|  | -0.092 | -1.023 | 0.562 |  |  |
|  | *8-talker – 4-talker* | | |  |  |
|  | 0.062 | 0.699 | 0.764 |  |  |
| **VerticalAmplitude** ~ noise + (1+noise\|PAIR)+(1+noise\|Verb),  Test = cumulative link |  |  |  | -0.363 | 0.398 |
|  | *8-talker – clear* | | |  |  |
|  | -168.70 |  | 0.132 |  |  |
|  | *4-talker – clear* | | |  |  |
|  | -94.64 |  | 0.434 |  |  |
| **MouthOpening** ~ noise + (1+noise\|PAIR) |  | | | 33.881 | < 0.001 |
|  | *8-talker – clear* | | |  |  |
|  | 13.3 | 0.567 | 0.838 |  |  |
|  | *4-talker – clear* | | |  |  |
|  | 2.10 | -0.108 | 0.994 |  |  |
|  | *8-talker – 4-talker* | | |  |  |
|  | 15.4 | 0.909 | 0.638 |  |  |
| **LipMovement** ~ noise + (1+noise\|PAIR)+(1\|Verb) |  |  |  | 16.620 | 0.020 |
|  | *8-talker – clear* | | |  |  |
|  | -0.029 | -0.220 | 0.974 |  |  |
|  | *4-talker – clear* | | |  |  |
|  | -0.059 | -0.472 | 0.885 |  |  |
|  | *8-talker – 4-talker* | | |  |  |
|  | 0.030 | 0.260 | 0.963 |  |  |
| **PeakLipVelocity** + (1\|PAIR) |  |  |  | 5.112 | 0.078 |
| **SpeechAmplitude** + (1\|PAIR)+(1\|Verb) |  |  |  | 11.496 | 0.003 |
|  | *8-talker – clear* | | |  |  |
|  | 0.290 | 3.374 | 0.002 |  |  |
|  | *4-talker – clear* | | |  |  |
|  | 0.123 | 1.316 | 0.387 |  |  |
|  | *8-talker – 4-talker* | | |  |  |
|  | 0.167 | 1.820 | 0.164 |  |  |
| **FundamentalFrequency** ~ noise + (1\|PAIR)+(1\|Verb) |  |  |  | 1.685 | 0.431 |
| **Modality** ~ noise + (1+noise\|PAIR)+(1+noise\|Verb),  Test = cumulative link |  |  |  |  |  |
|  | *8-talker – clear* | | |  |  |
|  | 1.813 |  | 0.150 |  |  |
|  | *4-talker – clear* | | |  |  |
|  | 0.248 |  | 0.772 |  |  |

Supplementary Table 3. Mixed model information for all main effects analyses. Final model structure, including random terms, and (when alternative) distribution family are given in the first column. Statistics for the model comparisons (final versus null) are provided for all models. For significant model comparisons, we also provide the effect estimate (**β**), as well as the associated t-ratio and p-value for each comparison, as computed using the emmeans package. Note that for the two MCMCglmm tests (i.e. vertical amplitude and modality), we provide the posterior mean and pMCMC values, as these tests do not utilize model comparisons, but directly assess the model terms.

| **Pair** | **Item** | **Stroke 1** | **Stroke 2** | **Stroke 3** | **Additional Function** |
| --- | --- | --- | --- | --- | --- |
| 77 | dippen (dip) | Repeated dipping with left hand | hand to mouth, eating | dipping again | elaboration |
|  | rijden (drive) | arms outstretched, moving hands up and down ( steering ) | larger movement, turning |  | repetition |
|  | jojoen (yo-yo) | hand moves up and down | same motion repeated |  | repetition |
| 5 |  |  |  |  |  |
|  | gooien (throw) | light toss straight up in the air | throw away to side |  | repetition |
| 13 |  |  |  |  |  |
|  | slingeren (swing) | pulls self up onto rope | continues to swing to the side |  | elaboration |
|  | kietelen (tickle) | starts tickling motion in front of self (other-directed) | self-directed tickling gestures |  | repetition |
| 20 |  |  |  |  |  |
|  | slingeren (swing) | moves in one large forward swing | continues swinging side to side |  | repetition |
|  | verbinden (connect) | pinches together two objects with fingers | presses together with palm of left hand | | elaboration |
| 59 |  |  |  |  |  |
|  | afgieten (pour/drain off) | stirring motion | pouring motion |  | elaboration |
|  | drijven (float) | swimming (breaststroke) | arms to sides, floating |  | elaboration |
|  | vangen (catch) | throwing, hands remain out | hands pull back to catch |  | elaboration |
|  | wijzen (point) | one heavily marked deictic, followed by hold | several rapid deictics after |  | repetition |
|  | dippen (dip) | several dipping motions | taking a bite |  | elaboration |
|  | vlechten (braid) | gestures at hair | depicts braiding (stacking motion with hands, while pinching) | moves hands back to hair | elaboration |
| 60 |  |  |  |  |  |
|  | kietelen (tickle) | localized tickling gesture (chest) | changes locations, moves body | | repetition |
| 76 |  |  |  |  |  |
|  | beklimmen (climb) | uses hands to outline mountain | climbing gesture moving up the mountain space | | elaboration |
|  | Verbinden (connect) | moves right hand to left, both fists as if holding something | pulls left away and then back to right | | repetition |
|  | Krassen (scratch) | makes scratching motion with palm flat | repeats the same motion with bent fingers | | repetition |
|  | Steken (stab) | one pronounced stab motion | two quicker stabs |  | repetition |
|  | Schrobben (scrub) | scrubs with left hand on right arm | scrubs chest |  | repetition |
|  | Timmeren (hammer) | hammers in front | hammers to the side |  | repetition |

Supplementary Table 4. Overview of gesture functions within a single communicative attempt.
